# Atypical Integration of Motion Signals in Autism Spectrum Conditions

**DOI:** 10.1371/journal.pone.0048173

**Published:** 2012-11-20

**Authors:** Caroline E. Robertson, Alex Martin, Chris I. Baker, Simon Baron-Cohen

**Affiliations:** 1 Department of Psychiatry, Autism Research Centre, University of Cambridge, Cambridge, United Kingdom; 2 Laboratory of Brain and Cognition, National Institute of Mental Health, National Institutes of Health, Bethesda, Maryland, United States of America; University of British Columbia, Canada

## Abstract

Vision in Autism Spectrum Conditions (ASC) is characterized by enhanced perception of local elements, but impaired perception of global percepts. Deficits in coherent motion perception seem to support this characterization, but the roots and robustness of such deficits remain unclear. We aimed to investigate the dynamics of the perceptual decision-making network known to support coherent motion perception. In a series of forced-choice coherent motion perception tests, we parametrically varied a single stimulus dimension, viewing duration, to test whether the rate at which evidence is accumulated towards a global decision is atypical in ASC. 40 adult participants (20 ASC) performed a classic motion discrimination task, manually indicating the global direction of motion in a random-dot kinematogram across a range of coherence levels (2–75%) and stimulus-viewing durations (200–1500 ms). We report a deficit in global motion perception at short viewing durations in ASC. Critically, however, we found that increasing the amount of time over which motion signals could be integrated reduced the magnitude of the deficit, such that at the longest duration there was no difference between the ASC and control groups. Further, the deficit in motion integration at the shortest duration was significantly associated with the severity of autistic symptoms in our clinical population, and was independent from measures of intelligence. These results point to atypical integration of motion signals during the construction of a global percept in ASC. Based on the neural correlates of decision-making in global motion perception our findings suggest the global motion deficit observed in ASC could reflect a slower or more variable response from the primary motion area of the brain or longer accumulation of evidence towards a decision-bound in parietal areas.

## Introduction

Autism Spectrum Conditions (ASC) are characterized by superiority in visual perception when task demands emphasize the perception of local elements, or deficits when task demands emphasize global elements [1]–[8]. Colloquially, such a perceptual style evokes the description of the autistic visual experience as “seeing the trees, but not the forest” [9]. The origins of this pattern of autistic visual perception are unclear. Here, we focus on one prominent example of a global visual deficit, coherent motion perception, with the aim of characterizing the mechanisms mediating the integration of local motion signals towards a global percept in ASC.

**Figure 1:**
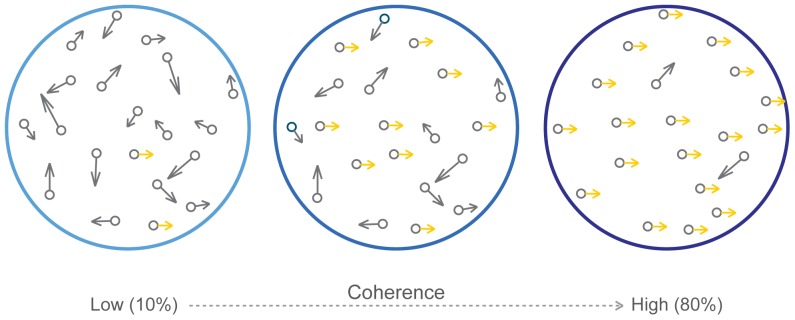
Coherent motion perception display. The coherent motion display contains a set of moving dots, a fixed proportion of which are moving in a coherent direction (e.g. 0° in the figure above, in gold), while the rest are randomly replotted. When this proportion (“coherence level”) is high, task difficulty is low.

In a traditional motion coherence task, participants are shown a display of moving dots on a computer screen. Frame by frame, a certain percentage of the dots are replotted in a constant direction, while others are randomly replotted (Figure 1). The more dots moving in a constant direction, the greater the coherence. Participants are asked to report the “general direction of motion” in the display, given two alternatives (e.g. “left” or “right”). An everyday example of this is the ability to judge the direction in which the wind is blowing a collection of leaves on the pavement. Each individual leaf is likely to have an unstable motion vector that, alone, cannot describe the overall direction of motion. However, our visual system widely samples and integrates these individual motion vectors towards an answer that optimally describes their common direction, their “coherent” or “global” motion.

Numerous studies have pointed to a deficit in coherent motion perception in ASC. Spencer et al. (2000) were the first to examine this task in an autistic population, finding that perceptual decisions about the direction of motion of moving dots were less accurate in ASC compared to control participants as the ratio of signal (coherently moving dots) to noise (randomly moving dots) was decreased. The authors reported coherent motion perception thresholds in ASC that were 45.6% higher than those of controls across a wide range of ages. At least six studies to date have replicated this coherent motion perception deficit in ASC [10]–[15], making it one of the most highly replicated findings in the literature on visual perception in ASC.

The primary aim of our study was to investigate whether coherent motion perception is categorically atypical in ASC, or simply exhibits a different dependence on the stimulus parameters that facilitate the integration of motion signals towards a global percept. More than a test of motion perception, coherent motion paradigms probe the ability to integrate local samples of perceptual evidence (individual motion signals) over space (the expanse of the display) and time (the duration of the stimulus) towards an aggregate percept. A wide range of stimulus parameters influence the rate of integration (e.g. dot contrast, density, size and speed). In the current study we investigated whether the rate at which motion signals are accumulated is atypical in autism by selectively titrating one specific parameter, stimulus duration.

Although the neural basis of the proposed atypicality in coherent motion perception in ASC is unknown, the decision-circuit underlying coherent motion perception has been extensively studied and modeled in the macaque. At a neural level, two brain areas are thought to support decision-formation: the primary motion area (middle temporal area or MT), responds to moment-by-moment motion signals in the environment, while neurons in the banks of the lateral intraparietal sulcus (LIP) aggregate these signals until sufficient evidence to make a perceptual decision has been collected [16], [17]. LIP response increases throughout the accumulation period (stimulus viewing duration), and culminates in a stereotypical plateau at the end of the integration period, which is thought to represent the arrival at a decision-threshold [18].

This bounded diffusion model of neural activity provides a compelling framework in which to consider autistic perceptual experience. Theories of ASC largely attribute the proposed motion processing deficits to either atypical processing of primary motion signals (e.g. “dorsal stream vulnerability” or “magnocellular” theories) [10], [11] or reduced integration of these signals (e.g. [19]) (see Milne et al. for review [20]). Understanding the effect of stimulus duration on performance can help distinguish between these possibilities. A categorical deficit in processing coherent motion would predict that individuals with ASC are unable to make global motion decisions, even when given extended integration time. Such performance could result from noisy primary motion signals, the sum of which does not reflect the accurate global motion direction even after extensive sampling (atypical primary motion processing), or reliable inconsistencies in integrating stable motion signals towards a decision-bound (unreliable decision formation). However, a deficit specific to the efficiency of motion integration predicts a distinct behavioral signature: recovery under longer stimulus durations (and therefore sensory integration times). Because motion signals accumulate additively over the time allotted for sensory integration, coherent motion perception thresholds are known to improve with approximately the square root of duration [21].

The secondary aim of our study was to address an apparent disagreement in the current literature on coherent motion perception in ASC. Alongside the numerous replications of a deficit in coherent motion perception in ASC, four failures to replicate have also been reported [22]–[25]. Since a deficit in this basic aspect of perception would have significant implications for learning and functioning, it is vital to understand why some studies have confirmed it and others have not. Considerable variation between the stimulus parameters used in previous studies prohibits a characterization of coherent motion perception in ASC by mere literature review (see Supporting Information, Table S1). However, as noted above, if coherent motion perception in ASC exhibits increased dependence on the stimulus parameters that facilitate integration, it is possible that confirmation of a deficit would depend on the parameters used in a particular study.

By examining global motion perception in people with and without ASC across a range of viewing durations, we find that the temporal dynamics underlying coherent motion perception in ASC are atypical. Autistic participants display a deficit in coherent motion perception judgments during short accumulation times, but their performance is restored as stimulus duration is lengthened. This argues that the global perceptual decision-making circuit is intact in ASC, but is supported by different dynamics.

## Materials and Methods

### Subjects

40 age-matched adult participants (20 high-functioning ASC) participated in our study. Cases met international criteria for ASC according to DSM-IV [26], as judged by specialist clinicians at the National Institutes of Health or a recognized clinic in the United Kingdom. All participants reported normal or corrected to normal vision. Individuals with Attention Deficit Hyperactivity Disorder were excluded from participation. The Autism Diagnostic Observation Schedule (ADOS) [27] was collected on a subset of ASC participants (n = 14), as was the Performance subscale of the Wechsler Abbreviated Scales of Intelligence (WASI) [n = 19 (ASC), n = 14 (Controls)] [28] (Table 1). Participants were recruited from the University of Cambridge's Autism Research Centre volunteer database (http://www.autismresearchcentre.com) or the Washington D.C. metropolitan area. No effect of testing location was observed on the pattern of our results. This study was approved by the University of Cambridge Psychology Review Board and the National Institute of Health Review Board. Written consent was obtained from all participants and/or their parent/guardian, in accordance with a protocol approved by the University of Cambridge Psychology Review Board or the National Institute of Health Review Board, as appropriate.

**Table 1:** Psychometric Data.

		N	Std. Dev	Mean*
Controls	Age (years)	20	3.45	24.65
	Performance IQ (T-score)	14	8.23	124.00
ASC	Age (years)	20	11.68	30.26
	Performance IQ (T-score)	19	18	111.89

* No significant differences were observed between the two groups in age or IQ (p>0.05).

### Stimuli

Participants performed three blocked versions of the classic forced-choice motion discrimination task [29], manually indicating the global direction of motion (right or left) of a random-dot kinematogram (RDK) containing 150 dots (dot diameter: 0.04 degrees). Viewing distance from the center of the screen was fixed using a chin rest (57 cm) and a fixation point was set 7° degrees of visual angle below the center of the stimulus aperture to align all participants' view of the stimuli. A peripheral fixation point was chosen to reduce the effect of coherent motion on eye movements. All testing took place in a dark room.

Performance was assessed using the method of constant stimuli. Participants completed twenty-one trials at each of seven different coherence levels. At each coherence level, a fixed percentage of dots were moving in a coherent direction for the duration of the trial. The levels of coherence (2%, 4%, 6%, 15%, 20%, 30%, 50%) were chosen to straddle perceptual thresholds at multiple durations. This wide array of coherence levels enabled us to carefully measure performance across the expanse of the psychometric function. An additional coherence level of 75% was measured for a subset of participants (n = 31, 13 ASC), to probe performance at a coherence level beyond which performance was expected to have reached ceiling.

The RDK patch of white dots (1.85 dots/deg^2^, displaced at a speed of 5.0 deg/s) appeared in an aperture on the LED monitor, the diameter of which measured 9° in visual angle. Coherence in the display was defined by the proportion of dots moving in the global direction of motion, rather than the variance of the motion vector of assigned to any one dot. On each frame (duration 16.6 ms), one third of the total set of dots was replotted, either in the direction of motion (at the speed of motion) or assigned a random position. The probability that a dot was shifted in the global direction of motion, as opposed to randomly replotted, was determined by the level of coherence in the display. (See references for previous descriptions of this noise paradigm [30], [31]). This limited lifetime paradigm precluded the possibility of participants tracking individual dots to form the global decision, as noted by previous reviews in the literature [32], [33]. Different viewing durations were created simply by increasing the number of frames in which dots were plotted; dot lifetime remained identical across viewing durations.

Coherence level (motion strength) and dot direction were randomly chosen on each trial. Stimulus duration was held constant in each block, but varied between the three blocks, always beginning with the 1500 ms duration block, followed by counterbalanced presentation of the 400 ms and 200 ms blocks. These durations ranged from 1500 ms, a long viewing duration at which motion evidence is thought to be saturated, to 200 ms, a duration at which accumulated motion evidence is just at the cusp of a decision threshold, as suggested by single-cell recordings in macaque [34]. An intermediate duration of 400 ms was also tested to examine the evolution of performance across decreasing integration times.

Stimuli were presented using PsychToolbox (www.psychtoolbox.org) [35] on a MacBook Pro running Mac OSX (resolution: 1440×900, refresh rate: 60 Hz). Stimuli scripts were adapted from the Shadlen lab's original MATLAB code (www.shadlen.org/Code/Matlab).

### Procedure

Prior to the experiment, participants were introduced to the task through both verbal description and a demonstration session at the highest level of coherence and longest viewing duration (1500 ms). The task was likened to watching a group of leaves glimmering on a tree in order to judge in what direction the wind was blowing.

**Figure 2:**
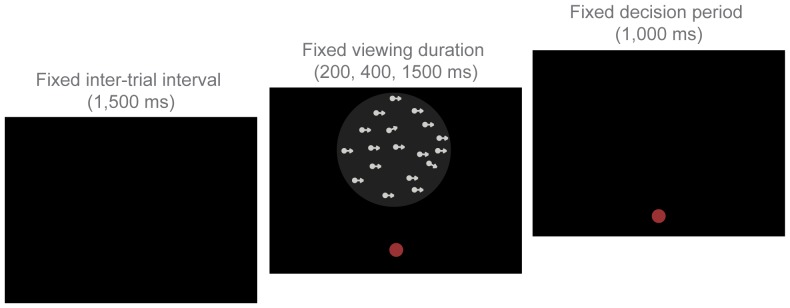
One trial of the motion coherence task. Participants manually indicated the “general direction of motion” of 150 white dots in a central aperture above a fixation point. Viewing duration varied between blocks (200 ms–1500 ms), but the decision period remained constant regardless of viewing duration.

During each trial, a patch of dots would appear on the screen within the viewing aperture for a fixed stimulus duration (e.g. 1500 ms), after which the screen would become blank and participants indicating the “general direction” in which the white dots were moving by key press. Participants were instructed to delay their response until after the stimulus had been removed from the screen, and premature responses were removed from the analysis so that responses reflected complete integration of the available motion signals. During the response period, participants were allotted a 1 s interval to make their decision, followed by a 1.5 s delay, regardless of stimulus duration. A red fixation point was present on the screen during the entire trial, even during the response period (Figure 2). Participants were instructed to fixate on this point throughout the task. The entire session took approximately 30 minutes to complete, with breaks inserted between each task.

### Analysis

All data were analyzed with custom scripts written in MATLAB (Mathworks, Inc., Natick, Massachusetts). Psychometric functions and motion coherence thresholds (82% correct) were evaluated using the Psychtoolbox extension for Matlab to fit individual averages of correct and incorrect responses, weighted by the number of responses at each coherence level, to a Weibull function.

To ensure participants included in the analysis were able to perform the task, participants were only considered for analysis if they were able to detect the general direction of motion (accuracy >88% correct) at the two highest coherence levels during trials enduring for the longest possible presentation time (1500 ms) (excluded n = 5, 2 ASC). This conservative method of assessing task performance meant that all participants included in the analyses were comfortable with the task instructions and making global motion judgments. Setting minimal constraints on the data in fitting to this function, we opted not to force the fit through 100% accuracy at 100% coherence. Thus, all individual thresholds are derived from fits to participants' own accuracy scores, without any artificial data points. As viewing duration decreased, an absolute threshold of 100% was given to any participant whose threshold met or surpassed 100% coherence, as estimated by the Weibull fit (n = 3 ASC). Analyses were additionally performed on the raw data, comparing the accuracy scores of each group across coherence levels. Participants whose 75% correct coherence thresholds were at ceiling were excluded from all correlational analyses performed at the 200 ms viewing duration (n = 2 ASC).

## Results

In examining the construction of a global percept in ASC, we aimed to determine whether atypical global perception found in ASC is attributable to deficits in visual integration. Holding all other parameters constant, we hypothesized that controlled titration of available sensory evidence would expose the minimum quantity of information necessary to make an accurate global motion judgment. We additionally aimed to explore the relationship between performance on our motion integration task and a diagnostic measure of ASC, the Autism Diagnostic Observation Schedule (ADOS).

### Atypical integration of motion signals in ASC

**Figure 3:**
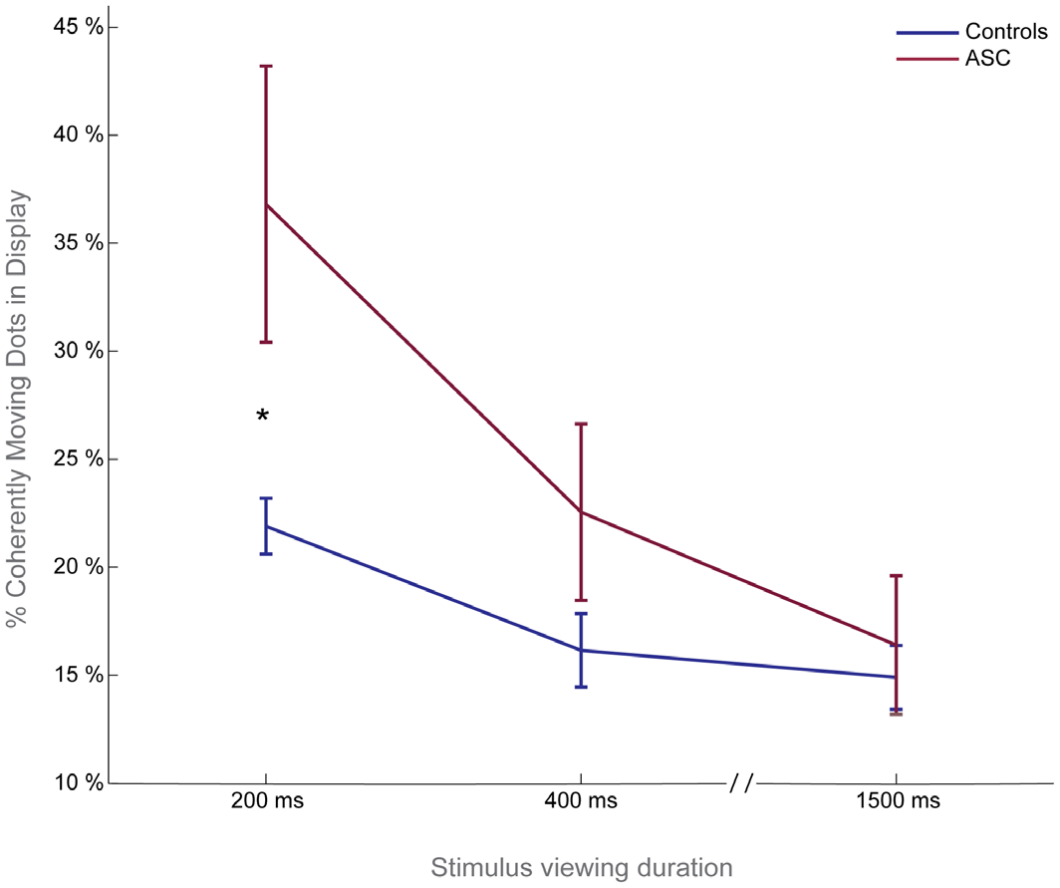
Coherent motion perception deficit in ASC as viewing duration is limited. Group coherent motion perception thresholds (+/−1 SE) are shown here. Coherent motion perception thresholds are significantly elevated in ASC at the 200 ms viewing duration. Longer viewing durations restore coherent motion perception, pointing towards atypical accumulation of motion signals in ASC.

Compared to controls, participants with ASC made significantly less accurate judgments about the coherent direction of motion present in the display at the shortest viewing duration. Increasing the viewing duration of the display improved coherent motion perception in both groups, but much more so in the ASC group, ultimately restoring coherent motion perception thresholds to the level of control performance (Figure 3).

We used a repeated-measures ANOVA to examine the change in coherent motion perception thresholds (82% correct, as estimated by fitting performance data to a Weibull curve) of individuals in each diagnostic group across viewing duration times. A significant interaction was found between stimulus duration time and diagnosis (F(2,76) = 4.054, p<0.021), indicating that controls and those with ASC are differentially affected by changes in viewing duration (Figure 3). Performance was significantly modulated by duration in both groups (F(2,76) = 17.506, p<0.001), and there was no main effect of diagnosis (F(1,38) = 3.696, p<0.062).

In order to characterize the interaction between stimulus duration and diagnosis, we performed a series of post-hoc statistical analyses of individual coherent motion perception thresholds at each viewing duration. This analysis revealed significantly higher perceptual thresholds in the group of participants with ASC only at the shortest viewing duration (200 ms, t = −2.373, p<0.23). ASC and control performance on the 400 ms and 1500 ms blocks were not significantly different (t = −1.489, p<0.145 and t = −0.445, p<0.658, respectively) (Figure 3). Group differences in the steepness parameter of the Weibull fits were not observed at any viewing duration (p>0.201), indicating that the slopes of the psychometric functions of individuals with and without ASC were comparable.

We also characterized performance in each group without fitting individual data points to a Weibull function. This analysis allowed us to explore individual components of the psychometric function which implicitly contribute to determining individual thresholds, such as the slope of the function, maximum and minimum accuracy, and the range of individual performance points at any level of coherence, and to remove any constraints imposed by the Weibull fit. Towards this end, a repeated measures ANOVA was computed on the raw behavioral data (across coherence levels and between groups). This analysis indicated that the group difference found in coherent motion perception thresholds at the 200 ms viewing duration could be mapped onto differences in the psychometric functions of each group.

**Figure 4:**
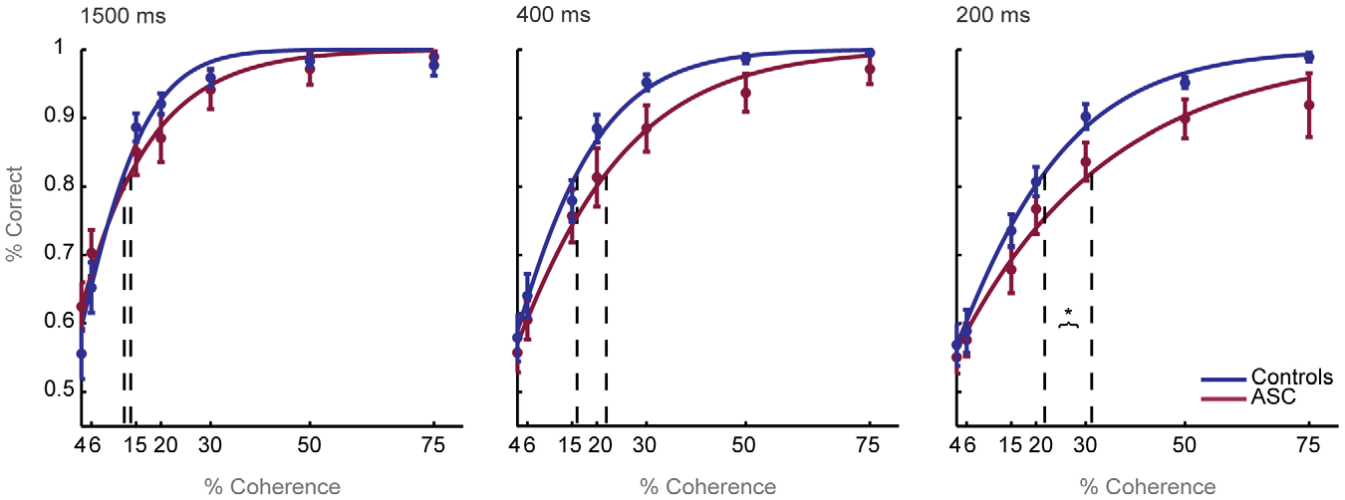
Performance across levels of coherence is offset in ASC at short viewing durations. Average percent correct at each coherence level (+/−1 standard error) is plotted along side a Weibull curve that is fit to group average performance (for illustrative purposes) for the three viewing durations. Dashed lines mark each group's 82% correct coherent motion perception thresholds. At short viewing durations, the ASC group produces significantly accurate responses across coherence levels.

Specifically, performance in the ASC group was consistently offset across all levels of coherence (Figure 4). In the 200 ms viewing duration, the ANOVA revealed a significant effect of group (F(1,38) = 4.633, p<0.038). This effect was observed at a trend-level at the 400 ms viewing duration (F(1,38) = 2.843, p<0.1), but completely absent at the 1500 ms viewing duration (F(1,38) = 0.163, p<0.689), indicating that the offset in performance observed in the ASC group was specific to short viewing durations. In both groups, performance was significantly modulated by coherence level at the short viewing duration (F(6,228) = 87.795, p<0.001), but no interaction of group by coherence level was observed (F(6,228) = 0.343, p<0.913), indicating that the dependence of performance on the coherence level in the display was comparable between autistic and control individuals.

Finally, in each group, performance reached ceiling at the two highest coherence levels (50% and 75%) at all viewing durations, as assessed by one-sample tests comparing performance to 95% accuracy (p>0.05). This indicates that both groups were able to judge highly coherent motion at all viewing durations. Performance at these high coherence levels did not differ significantly between groups (p>0.05), indicating that there were no differences in the ability to detect highly coherent global motion or attentional engagement with the task.

### Coherent motion perception deficit correlates with autistic symptomatology

**Figure 5:**
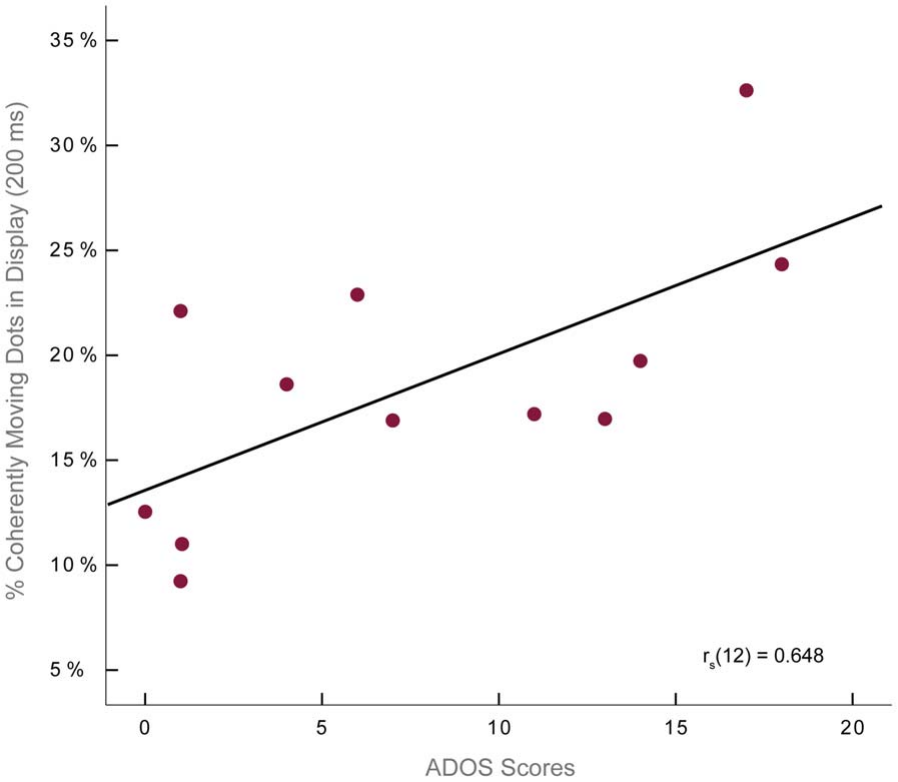
Coherent motion perception deficits correlate with measures of autistic symptom severity. A correlation was found between individual coherent motion perception thresholds at the shortest viewing duration and autistic symptoms, as measured by individuals' total scores on the Autism Diagnostic Observation Schedule (ADOS).

We also found an association between atypical visual integration and symptom severity in ASC. The deficit we report in coherent motion perception at the shortest viewing duration correlated strongly with autistic symptomatology. ADOS scores, specifically the combination of both subtests (social and communication), measured in the clinical group correlated with participants' 75% correct perceptual thresholds at the shortest viewing durations (Spearman's r_s_(12) = 0.648, p<0.023) (Figure 5). All correlational analyses were performed with individuals' 75% correct coherent motion perception thresholds to allow for more variability between individual thresholds than was available at the 82% correct level, where many of the subset of individuals with ADOS scores approached ceiling performance. Although one study has reported a relationship between performance on biological motion perception tasks and variability in autistic symptomatology [25], a relationship between coherent motion perception and autistic symptomatology has not previously been documented. This finding suggests that atypical visual integration may intimately relate to symptom severity in the social-communication deficits that define ASC.

### Performance is not influenced by IQ

This deficit in motion integration does not relate to participants' general levels of intelligence. Spearman rank correlation coefficients revealed no significant association between spatial ability and coherent motion perception thresholds at any duration in either group, as assessed using the age-corrected Performance scores of the WASI (p>0.427, two-tailed). Additionally, as a covariate in our repeated-measures ANOVA, IQ produced no interaction with stimulus duration (p<0.137). Although contrary to the findings of Koldewyn et al. (2009) [25], these results replicate the four previous studies that failed to find a significant relationship between verbal or nonverbal IQ and coherent motion perception thresholds [14], [24], [36], [37].

## Discussion

We report a deficit in coherent motion perception in ASC when viewing duration is limited to a short integration period, which is restored at long viewing durations. These results demonstrate for the first time that motion perception is not inherently atypical in autism, but operates with different dynamics. The deficit in coherent motion perception appears to result from a deficit in visual integration, rather than inaccurate primary motion processing or an inability to integrate local motion signals towards a global percept: thresholds in ASC are differentially aided by lengthening the window of time during which participants are able to view the stimulus, and thus accumulate perceptual evidence.

### Neural hypotheses regarding atypical integration of motion signals in ASC

Following the bounded diffusion model of global motion decisions, a deficit in global motion perception specific to short viewing durations could theoretically arise from one of two scenarios: an elevated decision-threshold or a slower rate of evidence accumulation. An elevated decision-threshold would require more time to complete the formation of a decision variable, potentially resulting in deficits in performance at short viewing durations where accumulated motion evidence might not be sufficient to reach the decision-bound. However, an elevated decision-threshold in ASC would also result in a steeper psychometric function. Each coherence level (reflected along the x-axis of the psychometric function) reflects a different strength of motion signal, and therefore a different rate of evidence accumulation [17]. Thus, the difference between their aggregate signals increases exponentially with time. Raising the decision-bound therefore exponentially affects their relative points-of-crossing. Psychophysically, this would result in a sharp falloff in performance as the level of coherence decreases in the display [38]. Alternatively, a slower rate of accumulation would also lead to a deficit in performance at short integration periods, requiring a longer time to accumulate towards the decision-threshold. Our results are consistent with this second possibility. We find a general offset in the psychometric function across all coherence levels in the 200 ms condition but no difference in the slope of this function, suggesting that evidence accumulation in the autistic perceptual system approaches a similar decision-threshold as in controls, but at a slower rate or with more variability. An increase in signal variability would predict both higher thresholds and lower accuracy across coherence levels due to more contribution from neurons tuned to irrelevant motion directions during the formation of the global motion decision-variable in the ASC group [39].

These results have implications for theoretical as well as mechanistic accounts of motion processing abnormalities in autism. One such theory, the “dorsal stream vulnerability” theory, interprets coherent motion perception deficits in ASC to reflect atypical primary motion processing due to abnormalities in the dorsal visual stream, potentially originating as early as magnocellular projections from the retina [10], [11], [40]. The strong version of this theory, that local motion signals are inherently unstable or inaccurate in ASC, would produce raised direction uncertainty: global motion decisions would be less reliable regardless of the stimulus viewing duration. The weak version, that motion signals are simply weaker or slightly less stable in ASC, would decrease the rate of incoming information, so that global motion decisions would exhibit a stronger dependence on integration time. Our results could support a weak version of the “dorsal stream vulnerability” theory: a decreased rate of incoming information could arise from a weak or slightly more variable early visual signal.

A second prevailing theory of coherent motion perception deficits in ASC posits that the level of variability in motion signals in the autistic visual system might be similar to controls, but the process of integrating such perceptual information is atypical, particularly affecting perception of complex [19] and global [9] stimuli due to the tax they place on integrative processes. The neural basis of such “integrative” theories is less clear. The strong version of this theory, that neural integration is categorically dysfunctional in ASC, would imply a centralized deficit in integration, perhaps related to setting a decision-threshold during perceptual decision formation. The weak version of this theory, that neural integration is simply less efficient in ASC, could originate from undersampling of motion signals across the expanse of the aperture, as suggested by Dakin and Frith (2005) [32]. Our results certainly provide support for a deficit in integrating motion signals, but remind that integrating dynamic stimuli is a temporally evolving process and rests on the amount of time allowed for evidence accumulation. As such, even integration of “complex” perceptual signals may not be atypical in the autistic visual system, but simply slower to emerge [41].

One possibility as to the neurobiological origins of this atypical integration of motion signals in ASC relates to the local circuitry of the primary motion area, MT. One of the defining features of MT is its response to motion opponency: each MT neuron is tuned to prefer motion in a particular direction, and is suppressed by motion that is orthogonal this direction [42]. Additionally, a moving dot will suppress response to a dot with an orthogonal motion vector up to 300 ms after the dot is displayed [43]. The main purpose of these inhibitory interactions is to filter noise; a task for which MT is uniquely wired [44]. This function plays a large role in global motion perception, selectively enable only one, dominant motion signal to survive among many contradictory signals in a given area of visual space [42], [45]. Increased inhibition between neurons in MT could potentially explain the increased dependence on stimulus duration time observed in our data, producing a weak population signal, which would take longer to emerge and aggregate towards a global decision. However, this weaker population response would contain well-filtered noise, and therefore support accurate judgments given ample integration time.

### Previous studies of coherent motion perception in ASC

Our novel finding that atypical dynamics underlie the integration of motion signals in ASC may help to synthesize previous reports of coherent motion perception deficits in autism. We suggest that a thorough understanding of coherent motion perception in ASC, or any population, demands consideration of the dynamics underlying evidence accumulation: coherent motion perception thresholds might be restored or atypical given the same signal-to-noise ratio in a display, depending on the amount of time allowed for evidence accumulation. Apparent disagreement between previous studies in the literature is therefore to some degree unsurprising, since no study has systematically titrated the amount of evidence available for decision-formation.

One difficulty in synthesizing the current literature on coherent motion perception in ASC relates to participant characterization: all but three studies [25], [46], [47] involve participants who are below the age at which coherent motion perception thresholds are thought to converge to adult levels (10–11 years) [48]–[51]. This is a particularly vital concern for interpreting the behavior of individuals with a developmental disorder such as ASC, as each group is likely to include wide developmental variation between individuals, and children with ASC and controls are known to show different developmental trajectories in motion perception [51]. Interestingly, when one examines the three studies of participants past the age at which adult motion thresholds have developed, the two studies employing shorter viewing durations (300 and 200 ms, respectively) [37], [46] report deficits in individuals with ASC, while the one with a longer viewing duration (2,000 ms) does not [25] (See Supporting Information, Table S1). Again, although notable, comparisons of this nature should be treated with caution given the variation in other stimulus parameters contributing to integration strength. Interestingly, variation in such parameters that contribute to integration strength, such as frame duration and dot speed, have been shown to elicit atypical motion processing in other developmental disorders, such as dyslexia [52]–[54].

### Implications for autistic symptomatology

Atypical integration of motion signals may be a marker of autistic symptomatology. We found a striking correlation between ADOS scores and perceptual thresholds in our participants with ASC. This finding is particularly interesting from a developmental perspective. Among the earliest behavioral markers of ASC are failures to make use of dynamic social cues in the environment: atypical gaze-monitoring [55], proto-declarative pointing [56], and visual orienting [55], [57] are thought to underlie deficits in joint attention that characterize autism and the development of a theory of mind [58], [59]. The neural mechanisms that shape the accumulation of dynamic gaze cues are strikingly similar to those implicated in decision-making about coherent motion signals. Single-cell recordings in the macaque LIP during gaze cuing reveal the same accumulation-to-bound behavior as during accumulation of evidence during moving dot paradigms [60]. If this mechanism is atypical in ASC during a standard motion decision task, might it also be atypical during a motion discrimination task in which the stimuli are not dots, but eyes? Examination of the mechanisms of integration central to gaze-following behavior might inform our understanding of its etiology in ASC.

A second dynamic social processing deficit observed in ASC, upon which our results might cast light, is biological motion perception. Biological motion deficits have been widely reported in autism [25], [61]–[64]. However, it is reasonable to suspect that deficits in rapid temporal integration of perceptual information would tax motion perception in biological as well as non-biological paradigms for two reasons. First, biological motion stimuli are, themselves, composed of motion vectors whose net directions are rapidly and often incoherently changing: the swinging right arm of a walking man may be reaching forward while his left leg kicks behind. Second, it is not unusual for “noise” in biological motion stimuli to be composed of a layer of incoherent random dot kinematogram (RDK) dots, overlaid on point-light walker stimuli [25]. “Noise” then is increased by reducing coherence in the RDK display. Thus, it seems reasonable to suspect that deficits in coherent motion perception may relate to deficits in biological motion perception. Along these lines, it is interesting to note that performance on coherent and biological motion tasks have been shown to correlate in individuals with ASC [25], [37]. Furthermore, one of the few fMRI studies of biological motion in ASC, conducted by our group, found reduced activity in MT+, rather than traditional biological motion areas such as the STS, in ASC in response to directional judgments of point-light walker stimuli [65]. The relation between coherent and biological motion perception remains an open question, however: rather than searching for correlations between independently generated and ambiguously matched stimuli, it would be important to examine the relative degradation of performance on biological and coherent motion tasks as integration periods are reduced.

### Conclusion

In sum, these results lead to two hypotheses of the etiology of coherent motion perception deficits in Autism Spectrum Conditions. First, perceptual evidence may be aggregated at a slower rate, due to either imprecision in or a decreased amplitude of local motion signals, requiring a longer viewing duration to accumulate sufficient perceptual evidence. Alternatively, people with ASC may require longer integration times because the bound towards which evidence is accumulated is elevated, or, what is formalistically identical, the prior from which motion evidence is accumulated is set lower. The characteristics of psychometric functions in our ASC group best support the first hypothesis. Future research will attempt to tease apart these two alternatives using functional magnetic resonance imaging (fMRI), examining motion responses in the autistic MT and parietal cortex. We conclude that our results point to atypical integration of motion signals in ASC: individuals with ASC require more evidence to form an accurate global decision than controls.

## Supporting Information

Table S1 **Previous psychophysical studies on coherent motion perception in ASC.** To facilitate comparison across studies, the table is sub-divided into studies using comparable paradigms. Top: studies using 2AFC direction-discrimination paradigms. Highlighted windows indicate studies in which the age range did not include participants below the age at which coherent motion perception is thought to have matured to adult levels (10–11 years). Bottom: studies using other paradigms that require coherent motion perception, such as detecting a patch of rotational motion in noise.
(DOCX)
