# Supplementary material for: Atypical Integration of Motion Signals in Autism Spectrum Conditions
Source: PLoS One. 2012 Nov 20;7(11):e48173. doi: 10.1371/journal.pone.0048173 (PMC3502435; doi:10.1371/journal.pone.0048173)
Supplement: Table S1 — Previous psychophysical studies on coherent motion perception in ASC. To facilitate comparison across studies, the table is sub-divided into studies using comparable paradigms. Top: studies using 2AFC direction-discrimination paradigms. Highlighted windows indicate studies in which the age range did not include participants below the age at which coherent motion perception is thought to have matured to adult levels (10–11 years). Bottom: studies using other paradigms that require coherent motion perception, such as detecting a patch of rotational motion in noise. (DOCX) [file pone.0048173.s001.docx]

**Two-alternative forced choice direction discrimination tasks**

|  | | **Stimulus Duration (ms)** | **Dot Number** | **Density**  **(Dots / deg^2^)** | **Speed**  **(Deg/s)** | **Dot Size**  **(Visual degrees)** | **Sample Size**  **(ASC/**  **Con)** | **Fixation Point** | **Screen**  **Distance (cm)** | **Age Matched Groups** | **Method of Threshold Estimation** | **Mean Age in Years**  **(ASC: Con)** | **Results** |
| --- | --- | --- | --- | --- | --- | --- | --- | --- | --- | --- | --- | --- | --- |
|  | Current study | 200 - 1500 | 150 | 1.85 | 5 | 0.04 | 20 / 20 | yes | 57 | yes | constant stimuli | 30 / 24 | Deficit in ASC at short durations only |
|  | Koldewyn et al. (2010) | 2,000 | 200 | 2.2 | 4.5 - 9 | 4 pixels | 30 / 30 | no | 60 | yes | constant stimuli | 15.12 / 15.78 | NS, when IQ is a co-variate |
|  | Atkinson (2009) | 200 | 750 | 5.85 | 22.9 | 0.11 | 13 / 16 | ? | 50 | yes | constant stimuli | 30 / 26 | Deficit in ASC |
|  | Takarae et al. (2008) | 300 | 100 | 2.26 | 3.3 | 0.15 | 41 (ASC 1) / 36 (ASC 2) / 46 | yes | 27 | yes | staircase | 16.3 (ASC1) / 15.25 (ASC2) / 16.5 (Con) | Deficit in ASC group with language delay (trend in combined ASC group) |
|  | Milne et al. (2006); White et al. (2006)^b^ | 1010? | 300 | 2.1 | 7 | 0.1 | 23 / 23 | ? | ? | yes | staircase | 10.1 / 10.25 | Deficit in ASC subgroup, not in whole group |
|  | Del Viva et al. (2006) | 160 | 100 | 0.4 | 10 | 0.4 | 10 / 12 (Con 1) / 14 (Con 2) | ? | 57 | yes | staircase | 8.8 / 6.6 (Con 1) / 9.7 (Con2) | NS |
|  | Davis et al. (2006) | 220, 1000 | 100 | ? | 6.3 | ? | 9 / 9 | ? | 70 | yes | staircase | 12.3 / 11.9 | Deficit in ASC at long duration; no interaction with viewing duration |
|  | Pellicano et al. (2005); Pellicano & Gibson (2008)^a^ | 600 | 100 | 0.4 | 6.3 | 0.11 | 20 / 20 | ? | 50 | yes | staircase | 9.5 / 9.8 | Deficit in ASC |
|  | Milne et al. (2002) | 1010 | 150 | 0.3 | 8.8 | 1 pixel | 25 / 22 | yes | ? | yes | staircase | 11.7 / 11.6 | Deficit in ASC |

**Other tasks requiring coherent motion perception**

|  | | **Stimulus Duration (ms)** | **Dot Number** | **Density**  **(Dots / deg^2^)** | **Speed**  **(Deg/s)** | **Dot Size**  **(Visual degrees)** | **Sample Size**  **(ASC:**  **Con)** | **Fixation Point** | **Screen**  **Distance (cm)** | **Matched Groups** | **Method of Threshold Estimation** | **Mean Age in Years**  **(ASC: Con)** | **Results** |
| --- | --- | --- | --- | --- | --- | --- | --- | --- | --- | --- | --- | --- | --- |
| **Direction discrimination in an "odd one out" paradigm** | de Jonge et al. (2007) | no limit | ? | ? | ? | ? | 29 / 32 | ? | 50 | yes | staircase | 16.6 / 17.6 | NS |
|  | Spencer et al., (2000) | motion direction reverses every 330 ms | ?` | 4 | 5.8 | ? | 23 / 50 | ? | 50 | yes | staircase | 7-11 / 7-11  (means not provided) | Deficit in ASC |
| **Detection of a patch of coherent, rotational motion in a noisy motion display** | Tsermentseli et al., (2008) | 250 | ? | 4 | 5.8 | ? | 21 / 20 | yes | 50 | yes | staircase | 28.3 (ASC1) / 23.3 (ASC2) / 28.4 | Deficit in ASC |
|  | Spencer et al., (2006) | 250 | ? | 4 | 5.8 | ? | 25 / 15 | ? | 40 | yes | staircase | 13.5 / (ASC1) 12 (ASC2) / 11.7 | Deficit in ASC |

a. The data presented in Pellicano et al. (2008) are the same as in Pellicano et al. (2005)

b. Likewise White et al. (2006) present data previously reported in Milne et al. (2006)

Question marks indicate unreported or unclear stimulus parameters.

**Table S1. Previous psychophysical studies on coherent motion perception in ASC**. To facilitate comparison across studies, the table is sub-divided into studies using comparable paradigms. Top: studies using 2AFC direction-discrimination paradigms. Highlighted windows indicate studies in which the age range did not include participants below the age at which coherent motion perception is thought to have matured to adult levels (10-11 years). Bottom: studies using other paradigms that require coherent motion perception, such as detecting a patch of rotational motion in noise.
